# Fusion of Laser-Induced Breakdown Spectroscopy and Raman Spectroscopy for Mineral Identification Based on Machine Learning

**DOI:** 10.3390/molecules29143317

**Published:** 2024-07-14

**Authors:** Yujia Dai, Ziyuan Liu, Shangyong Zhao

**Affiliations:** College of Optical, Mechanical and Electrical Engineering, Zhejiang A&F University, Hangzhou 311300, China; daiyujia@zafu.edu.cn (Y.D.); liuziyuan@zafu.edu.cn (Z.L.)

**Keywords:** mineral classification, laser-induced breakdown spectroscopy, Raman spectroscopy, data fusion, machine learning

## Abstract

Rapid and reliable identification of mineral species is a challenging but crucial task with promising application prospects in mineralogy, metallurgy, and geology. Spectroscopic techniques such as laser-induced breakdown spectroscopy (LIBS) and Raman spectroscopy (RS) efficiently capture the elemental composition and structural information of minerals, making them a potential tool for in situ and real-time analysis of minerals. This study introduces an integrated LIBS-RS system and the fusion of LIBS and RS spectra coupled with machine learning to classify six different types of natural mineral. In order to visualize the separability of different mineral species clearly, the spectral data were projected into low-dimensional space through t-distributed stochastic neighbor embedding (t-SNE). Additionally, the Fisher score (FS) was used to identify important variables that contribute to the data classification, and the corresponding chemical elements and molecular bonds were then interpreted. The between-minerals difference in the feature spectral intensity of LIBS and RS variables could also be observed. After the minerals spectra were pre-processed, the relationship between spectral intensity and the mineral category was modeled using machine learning methods, including partial least squares–discriminant analysis (PLS-DA) and kernel extreme learning machine (K-ELM). The results show that K-ELM and PLS-DA based on the fusion LIBS-RS data achieved the highest accuracy of 98.4%. These findings demonstrate the feasibility of the integrated LIBS-RS system combined with machine learning for the fast and reliable classification of minerals.

## 1. Introduction

In the fields of mineralogy, metallurgy, and geology, it is critical and difficult to quickly and accurately identify minerals. The identification of different types of minerals often relies on extensive chemical analysis due to the similarity of their elemental composition and complex structure. Some high-precision techniques based on potassium dichromate, titration, and ethylenediaminetetraacetic acid capacity methods are time-consuming and require professional knowledge and operation skills [1]. Moreover, these methods are destructive and unsuitable for handling large-scale samples. Therefore, rapid and simple analytical methods are urgently needed to improve the efficiency of mineral identification.

Optical spectroscopy has provided an efficient tool for identifying the species, chemical composition, and elemental distribution of minerals [2]. Numerous analytical technologies frequently applied to mineral analysis include Fourier transform infrared spectroscopy [3], hyperspectral imaging [4], near-infrared spectroscopy [5], laser-induced breakdown spectroscopy (LIBS) [6,7], and Raman spectroscopy (RS) [8]. Among these techniques, LIBS and RS are two popular methods for studying the chemical elemental and molecular information of material composition and detecting ingredients, respectively. LIBS technology is regarded as the “future superstar of chemistry analysis” [9] with its excellent online and in situ multi-elemental analysis capabilities. It is used for the qualitative and quantitative analysis of chemical elements or simple molecular compounds within substances. RS technology can provide molecular vibrations, structural discernment, and molecular composition information. Both LIBS and RS have the advantages of high speed, low sample preparation, less destructiveness, and simple and safe operation [10,11,12]. However, the LIBS signal quality is significantly reduced by factors such as the self-absorption effect, matrix effect, environmental influence, and violent expansion of the plasma [9], whereas the lower signal-to-background ratio (SBR) in RS is mainly caused by weak scattering signal intensity and fluorescence interference [13]. These issues, coupled with the low representativeness of raw minerals consisting of inhomogeneous substances, will greatly degrade the quality of LIBS-RS spectra and challenge the effectiveness of traditional analytical models such as linear discriminant analysis (LDA). 

To improve the performance of qualitative and quantitative analysis in mineral identification, machine learning (ML) is used to effectively extract high-level fingerprint information from the spectra that is closely related to real attributes. ML methods use statistical models and algorithms to enable computers to search through huge amounts of data for patterns and then use these identified patterns to describe or predict new data. Ishikawa et al. designed an automated model using RS, incorporating pre-processing, principal component analysis (PCA) for dimensionality reduction, and artificial neural network (ANN) classification [14]. This RS-based model was tested on a public mineral dataset (RRUFF [15]), achieving an accuracy of 80.4%. Liu et al. enhanced the classification accuracy on the same dataset by employing convolutional neural networks (CNN) with RS, surpassing the baseline model and attaining the highest tested accuracy of 88.4–96.3% [16]. Harmon et al. utilized LIBS to identify mineral samples based on unique chemical fingerprints in the emission spectra, achieving a correct rate of over 90% in linear correlation analysis during mixed mineral experiments [7]. Other linear methods based on LIBS, such as linear discriminant analysis (LDA) and partial least squares–discriminant analysis (PLS-DA), have also proven successful in identifying the spectra of geological samples [17,18]. Recent studies using LIBS have addressed nonlinear problems caused by matrix effects and signal uncertainty by applying nonlinear models such as support vector machines (SVM), ANNs, and CNNs [19,20,21]. Nonlinear methods generally have better predictive performance than linear ones by extracting high-level features from the spectra. 

The fusion of spectroscopic techniques is a promising way to improve the accuracy of mineral analysis in addition to using state-of-the-art machine learning algorithms. It contains varying types of spectral information (such as atomic and molecular information) and offers more comprehensive information about elements related to mineral composition. Some typical fusion techniques reported in recent years are XRF-VNIR [22], VNIR-LIBS [23], VNIR-RS [23], and LIBS-RS [23,24,25,26,27]. Specifically, Khajehzadeh et al. [22] adopted XRF-VNIR to quantitatively identify the mineral content of the slurry and obtained better results than using XRF or VNIR baseline measurements. Jahoda et al. [23] evaluated the performance of VNIR-LIBS, VNIR-RS, and LIBS-RS for mineral classification. They demonstrated that deep learning could further increase prediction accuracy. However, it was not made clear how much the variables associated with the characteristic elements contributed to the prediction results. The fusion of LIBS and RS can be visualized in the plasma emission spectra and molecular bond vibration related to the same substance, respectively [25]. However, it should be noted that the aforementioned fusion measurement method uses separate spectrometers, which lengthens the measurement time and increases the operational complexity in practical applications [26,27]. Since LIBS and RS measurements can be carried out with the same laser and spectrometer [28,29], the integrated LIBS-RS system has the potential to retain the simplicity of spectroscopic measurement and guarantee reliable and accurate mineral analysis results.

In this work, we demonstrate the feasibility of using an integrated LIBS-RS system with a single spectrometer combined with machine learning for fast and reliable mineral classification. Our research goals are the following: (1) to evaluate if the fusion of LIBS and RS data provides superior classification performance compared to using LIBS or RS data individually and (2) to determine if combining fused LIBS-RS data with machine learning methods can further enhance classification accuracy. We obtained LIBS and RS spectra of six types of minerals: strawberry quartz, jasper red, apatite, cherry quartz, southern red agate, and cat’s eye. Additionally, we projected the high-dimensional spectral data into low-dimensional space using t-distributed stochastic neighbor embedding (t-SNE) to visualize the separability of different mineral types. Fisher score (FS) was employed to select important spectral lines. After pre-processing the mineral spectra, partial least squares–discriminant analysis (PLS-DA) and kernel extreme learning machine (K-ELM) were used to identify the mineral samples. The results indicate that the spectroscopic fusion approach is highly effective for mineral identification.

## 2. Results and Discussion

### 2.1. LIBS and RS Spectra

**Figure 1:**
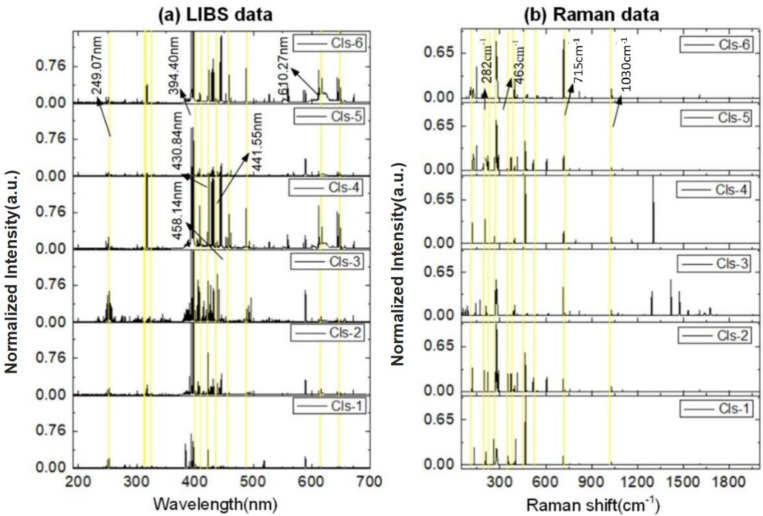
The mineral samples of processed LIBS spectra (**a**) and Raman spectra (**b**).

As can be seen from the LIBS-processed spectra shown in Figure 1a, certain elements exhibit high-intensity spectral lines in different kinds of minerals, such as Fe I (249.07 nm), Ca I (458.14 nm), Na I (588.99 nm), Ti I (430.84 nm), and Al I (394.40 nm, 441.55 nm). The intensity of some typical lines varies between different categories. For instance, the line intensity of Ca I (610.27 nm) is relatively high in Cls-4 and Cls-6. Due to the greater probability of Stokes processes as compared with anti-Stokes processes, it was chosen to obtain the Stokes part of RS. From Figure 1b, it can be seen that functional groups such as CO_3_^2–^ (715 cm^−1^), 282 cm^−1^ and Fe-O (463 cm^−1^) have high intensities in different types of Raman spectra [30]. The peaks above 1200 cm^−1^ in Figure 1b are attributed to higher-energy vibrational modes, such as the stretching vibrations of Si-O and Al-O bonds in the silicate framework of anorthoclase as well as bending OH vibrations in aerinite. This is consistent with similar feldspar minerals found in the RRUFF database, confirming the stability and reproducibility of these spectral features. The broadening observed in some of our Raman spectra is due to sample heterogeneity and the averaging of spectra from different regions. Additionally, the Raman spectra underwent data processing as described in Section 2.3, resulting in broadening that differs from typical Raman spectra. Two detailed tables presenting the emission lines of LIBS and RS can be found in Tables S1 and S2 (Supplementary Materials). Therefore, differences in the spectral intensity (LIBS measurement) and chemical bond (RS spectra) between different categories provide the feasibility for mineral identification. In order to make the process of mineral classification and key element identification easier to operate, the spectral data were input into machine learning models.

### 2.2. Mineral Classification

**Figure 2:**
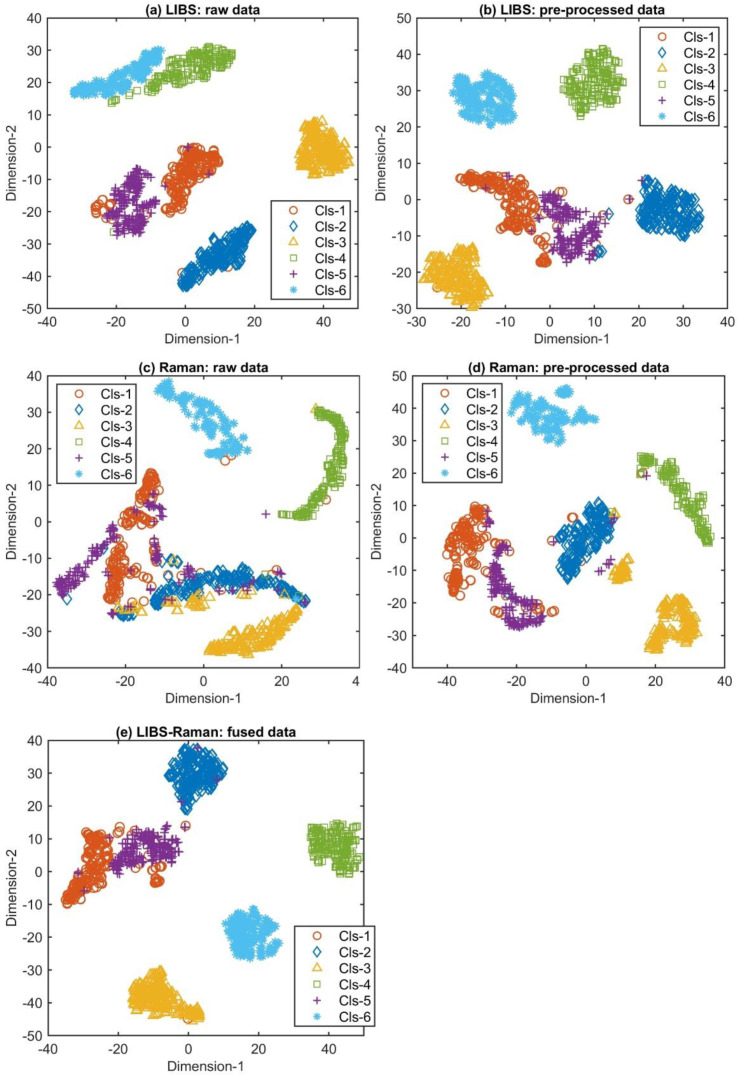
t-SNE visualization of mineral spectra: raw LIBS spectra (**a**), pre-processed LIBS spectra (**b**), raw Raman spectra (**c**), pre-processed Raman spectra (**d**), and fused spectra (**e**).

To visualize how various mineral classes can be separated, t-SNE was used to map the obtained spectra into a two-dimensional space, and the results are shown in Figure 2. In LIBS spectra (Figure 2), pre-processed Raman spectra (Figure 2d), and fused spectra (Figure 2e), it can be seen that most types of samples can be separated clearly. Moreover, the pre-processing improves the degree of distinction between Cls-4 and Cls-6 in LIBS spectra as well as Cls-1, 2, 3, and 5 in Raman spectra. Due to the similarity of the principal components of Rose Quartz and Southern Red Agate minerals, these two samples are spectrally similar. The Cls-1 (red) and Cls-5 (purple) clusters are comparably close by visual observation, which reveals the high degree of spectral similarity between Cls-1 and Cls-5. This suggests that data fusion improves the class separability of Raman spectra but may not significantly increase that of LIBS spectra. After obtaining effective classification results, we paid more attention to which variables were more important to the model prediction results. We adopted a simple FS method to intuitively reflect the importance of each variable and assess whether the corresponding components of these variables constitute the cause of mineral differentiation. The significant variables (wavelength and Raman shift) that help to distinguish different mineral categories can be identified by FS, as shown in Figures S2 and S3, and Table S3 (Supplementary Materials), respectively. These variables were determined based on the specific spectral characteristics observed across multiple sample positions and analytical repetitions [23,25,30]. After averaging the FS obtained from the training data of 50 repetitions, the variables with a high average FS were usually found to have a fairly larger contribution to the classification results.

### 2.3. Mineral Identification

Table 1 compares the average results of PLS-DA on the raw and pre-processed spectral data. In the training phase, the classification accuracy of LIBS and Raman data was improved from 92.63% to 94.10% and 90.36% to 93.34% by pre-processing, respectively. Moreover, pre-processing helped PLS-DA achieve a test accuracy of 95.61%, which is an improvement of approximately 1% over the result from the raw data. Also, the standard deviation of the 50 runs could be reduced after pre-processing.

**Figure 3:**
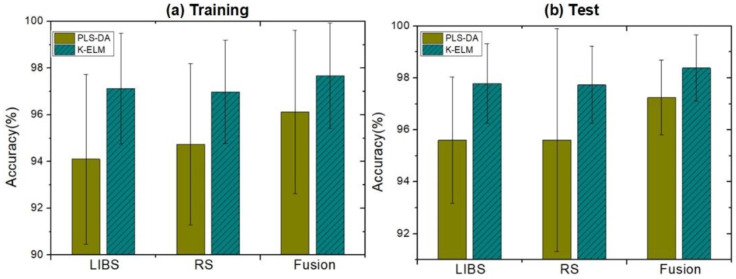
The average accuracy of the 50 repetition results of LIBS, RS data, and fusion data for training (**a**) and test set (**b**).

**Figure 4:**
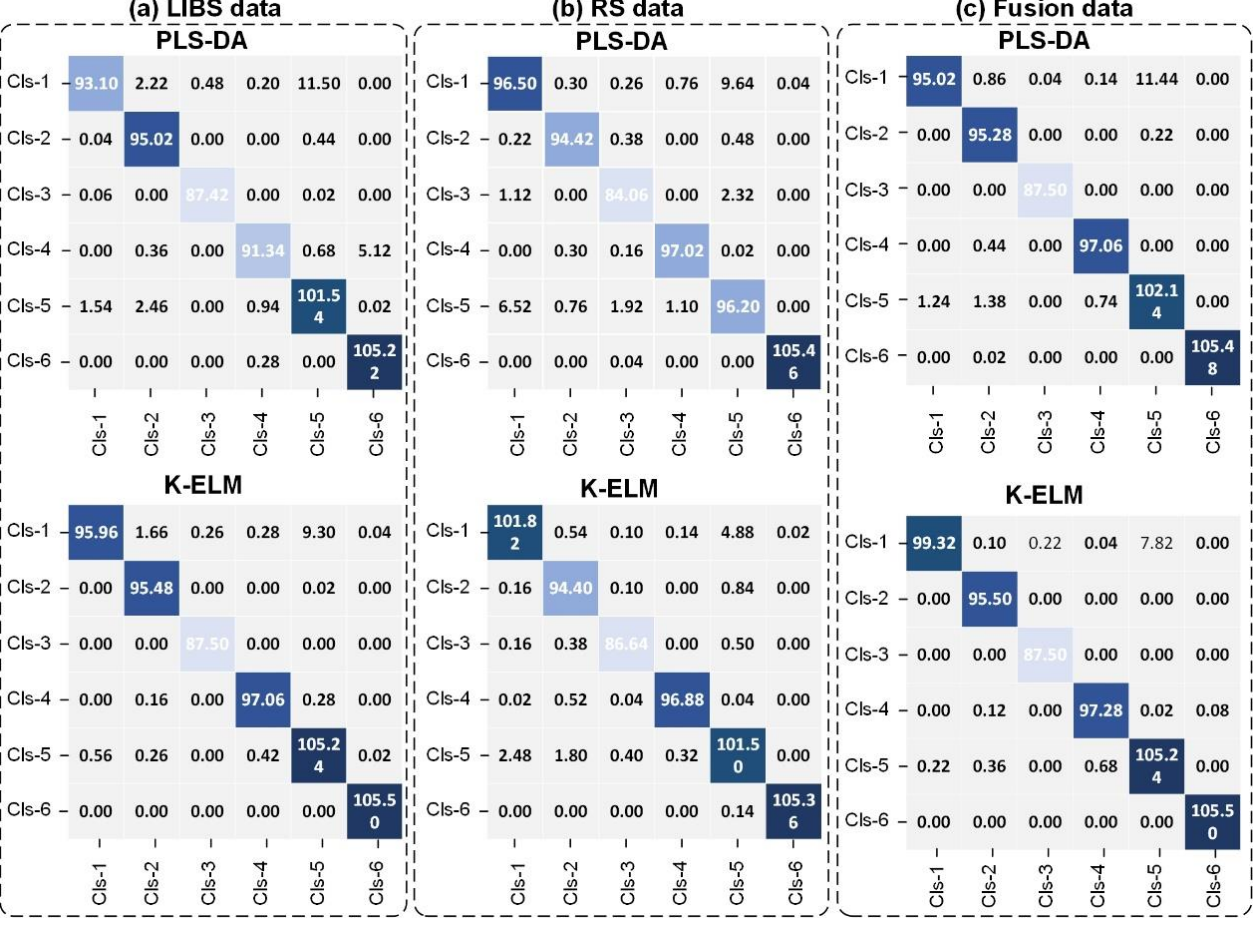
Confusion matrix of mineral classification using PLS-DA and K-ELM for (**a**) LIBS data, (**b**) RS data and (**c**) Fusion data.

We further improved the accuracy of mineral classification by using K-ELM and data fusion technologies. Figure 3 and Table 2 compare the results of different classifiers (PLS-DA and K-ELM) and measurement methods (namely LIBS, RS, and spectroscopic fusion). The error bar in Figure 3 represents the standard deviation of the 50 repeated experiments. K-ELM improved the classification accuracy of LIBS and RS data, reaching around 97.0% and 97.8% in the training and testing stages, respectively. Significantly, the spectroscopic fusion further increased the accuracy of mineral classification. The method was 0.5–1.5% more accurate than that of single measurements, with the highest test accuracy of 98.4% achieved when combined with K-ELM. Figure 4 displays the confusion matrix of the average value of test results, where the LIBS, RS, and LIBS-RS fusion data were classified using PLS-DA and K-ELM. For the K-ELM of fusion data, eight spectra that should have been assigned to Cls-1 were incorrectly classified to Cls-5. In Cls-4 and Cls-5, the number of wrongly identified spectra did not exceed one and two, respectively, while all spectra of Cls-2, Cls-3, and Cls-6 were perfectly correctly classified. These results are consistent with the visual distinction of various types of minerals in Figure 2e.

The basic components of minerals, rocks, and ores usually exist in coexisting assemblages. Some minerals have similar chemical compositions, such as Cls-3 and Cls-5, which cannot be identified with a high recognition rate using a single detection technology alone. Since LIBS and RS measurements can be performed using the same laser and spectrometer, the integrated LIBS-RS system can efficiently capture both the elemental composition and molecular structure information of minerals and reduce the uncertainty of signal, thus becoming a potential tool for in situ and real-time analysis of minerals. Generally, the models with high complexity (more parameters, like the K-ELM in this article) can deal effectively with nonlinear and complex data and yield good accuracy, but such models have poor interpretability. However, the model with good interpretability (PLS-DA) has low complexity and may not give satisfactory results when dealing with nonlinear and complex data. In fact, the accuracy is more of a concern than interpretability and variable importance in practical applications.

## 3. Experiment Methods

### 3.1. Mineral Samples

**Figure 5:**
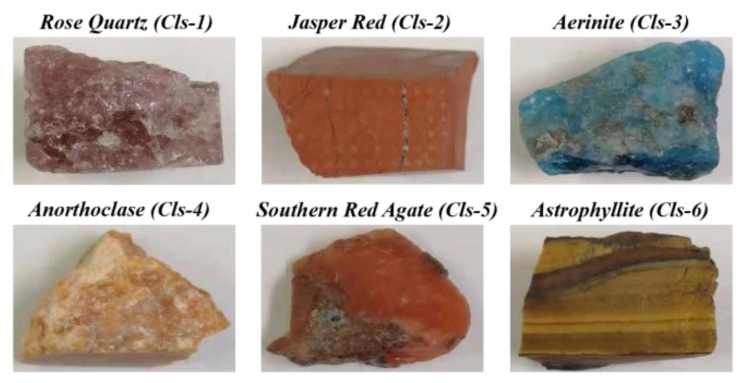
Mineral samples (Rose Quartz (Cls-1), Jasper Red (Cls-2), Aerinite (Cls-3), Anorthoclase (Cls-4), Southern Red Agate (Cls-5), and Astrophyllite (Cls-6)).

Figure 5 shows the six types of mineral samples used in this work: rose quartz (Cls-1, SiO_2_ with hematite inclusions, commonly used in gemology but not IMA-approved), jasper red (Cls-2, SiO_2_ with iron oxides), aerinite (Cls-3, Ca_4_(Al, Fe, Mg)_10_Si_12_O_35_(OH)_12_CO_3_·12H_2_O), anorthoclase (Cls-4, (Na, K)AlSi_3_O_8_), southern red agate (Cls-5, SiO_2_ with iron oxides and hydroxides), and astrophyllite (Cls-6, K_2_NaFe^2+^_7_Ti_2_(Si_4_O_12_)_2_O_2_(OH)_4_F). These samples were chosen to cover a variety of mineral types with distinct elemental and structural characteristics, which is essential for demonstrating the effectiveness of our LIBS-RS system combined with machine learning for mineral identification. Despite the obvious visual differences among the mineral samples shown in Figure 5, relying solely on visual inspection for mineral classification has significant limitations. Visual characteristics such as color and texture can vary within the same mineral type due to impurities and environmental factors. Therefore, visual inspection is not always reliable for accurate identification. The integrated LIBS-RS system provides a more precise method by analyzing the elemental and molecular composition of the minerals. This approach addresses the limitations of visual methods and ensures higher classification accuracy, which is crucial for practical applications in geological exploration and industrial mineral processing. Each mineral type was represented by eight samples, and the sample surface was not pre-treated. While the number of mineral samples of each type was limited to eight, we collected 25 spectra from different regions of each sample using both LIBS and RS techniques. Consequently, this resulted in a substantial dataset of 2400 spectra (2 measurement techniques × 6 mineral types × 8 samples per type × 25 spectra per sample), which provided a robust foundation for our analysis. Achieving high classification accuracy with this dataset highlights the initial effectiveness of the integrated LIBS-RS system combined with machine learning algorithms. However, we acknowledge the necessity of testing a larger number of samples from various localities and with different impurity compositions to fully validate the robustness and generalizability of our method. This will be a focus of our future research. In general, minerals contain a variety of cations and anions. For example, aerinite is a bluish-purple inosilicate mineral with the chemical formula Ca_4_(Al, Fe, Mg)_10_Si_12_O_35_(OH)_12_CO_3_·12H_2_O, which is chemically complex and includes carbonate-bearing silicate as the main inclusion. Unlike aerinite, jasper red is composed mainly of silicon dioxide (SiO_2_) with iron oxides (Fe_2_O_3_ and FeO) as the main inclusion, along with metal ions such as Mn^2+^, Fe^2+^, K^+^, and Ca^2+^.

### 3.2. Integrated LIBS-RS System

**Figure 6:**
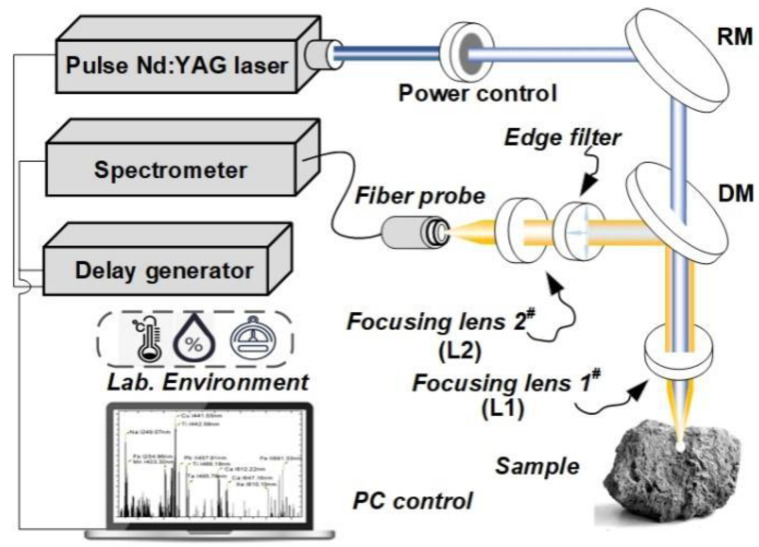
Schematic illustration of the time-gating LIBS-RS measurement system configuration (Reflection mirror, RM; dichroic mirror, DM; focusing lens #1, L1; focusing lens #2, L2).

**Figure 7:**
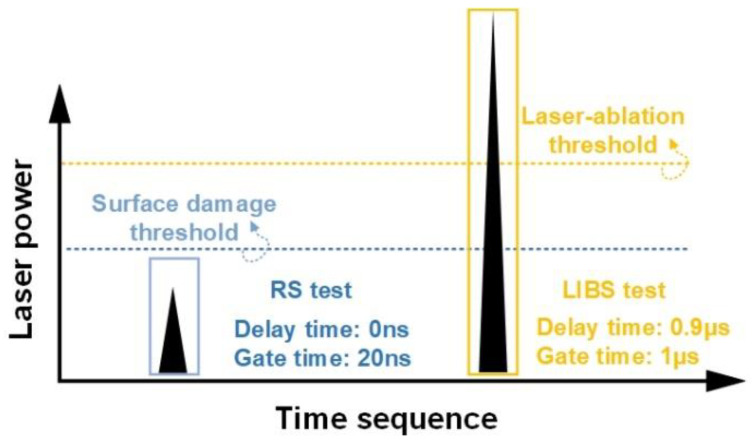
Schematic diagram of the spectra generation sequence of RS and LIBS (Raman spectroscopy, delay time 0 ns, gate time 20 ns; LIBS, delay time 0.9 μs, gate time 1 μs.) There is an order of measurement: first the Raman signal, then the LIBS signal.

Figure 6 shows the schematic diagram of the integrated LIBS-RS system, which consists mainly of a pulse laser source, a digital delay generator (DDG), a spectrometer, and peripheral optical components. For RS scattering excitation and LIBS sample ablation, a frequency-doubled Nd, i.e., YAG pulse laser (Beamtech, Dawa-200, wavelength 532 nm, pulse width 7 ns, repetition rate 1 Hz), was used. The output pulse energy was attenuated to 5 mJ (RS measurement) and 35 mJ (LIBS measurement) by an attenuation system (consisting of a half-wave plate and a thin-film polarized device), as shown in Figure 6 and Figure 7. The laser beam was perpendicular to the target surface. Specifically, it was focused onto the target via a 100 mm focal length lens L1, producing a spot of approximately 300 μm in diameter on the target’s surface. The coaxial polarized optical system is mainly composed of a dichroic mirror, polarizers, and L2 (f=50 mm), which collects Raman scattering spectra and LIBS spectra. The RS and LIBS spectra were recorded using a Czerny–Turner spectrometer (Andor, spectral resolution 0.05 nm) equipped with an intensity charge-coupled device (Andor, PI MAXII, ICCD, 1024 × 256 pixel). The Raman shift can be calculated by the equation $\left(1/\lambda_{0}-1/\lambda )\times 10^{7}({cm}^{-1}\right)$, where $\lambda_{0}=5$32 nm, and λ is the scattering wavelength. The region of interest is about 533.3–625.0 nm. The spectral ranges of RS and LIBS are 45–2000 cm^−1^ and 200–700 nm, respectively. The spectral range from 533.3 to 625 nm was chosen to encompass essential Raman shifts associated with the molecular vibrations and lattice modes characteristic of the minerals investigated. This selection optimizes the detection of distinctive spectral signatures crucial for mineral classification. We used a digital delay signal generator (Stanford Research, DG645) to control the delay time between the laser pulse and the spectral acquisition pre-trigger signal. Theoretically, the delay time can be set to 0 ns and 0.9 μs for RS and LIBS measurements, respectively. In the experiment, by optimizing parameters such as time delay, gate width, and laser power, we first collected the RS signal (laser power 5 mJ, no destruction of sample surface) and then the LIBS signal (laser power 35 mJ, micro-damage of sample surface). The schematic diagram of the RS and LIBS spectra generation sequence is shown in Figure 7. The mineral sample was stuck on a translation stage, giving a motion resolution of 10  μm and a travel range of about 30 mm. A 532 nm edge filter was added mainly to shield the intense Rayleigh scattered radiation while performing Raman measurements.

### 3.3. Spectral Data Processing

As shown in Figure S1 (Supplementary Materials), the processing workflow of raw spectra comprises several critical stages: (1) data dimensionality reduction using PCA and t-SNE; (2) baseline correction employing the least squares method; (3) noise removal via smooth filtering; (4) standardization using the minimum–maximum normalization method; (5) interpolation employing linear and polynomial methods; (6) utilization of the Fisher score (FS) to identify significant variables contributing to data classification, involving principal component selection and identification based on FS scores and spectral database, in which higher scores indicate characteristic spectra lines that are more crucial for model classification; and (7) following the preprocessing of mineral spectra, the modeling of relationships between spectral intensity and mineral categories using machine learning techniques including partial least squares–discriminant analysis (PLS-DA) and kernel extreme learning machine (K-ELM).

For each mineral sample (a total of 68 samples), 25 spectra from different regions, with 14,950 and 1930 variables based on LIBS and RS measurements, respectively, were acquired. Figure 1a,b show the average LIBS and Raman spectra of different types of mineral samples, respectively. The detailed data of the significant Raman peaks shown in Figure 1b are provided in Table S1 (Supplementary Materials). The raw spectra were first pre-processed with peak identification, baseline removal, peak area calculation, and standard normal variate (SNV). Among them, the SNV process is essentially a mathematical transformation to scale variable values to a unified interval, usually between [0,1]. After pre-processing, 363 and 97 variables highly correlated with chemical elements were fed into PLS-DA and K-ELM for data classification, respectively. Important variables (displayed as yellow bars) that contributed to data classification were selected and are shown in Figure 1. PLS-DA is a chemometrics method for analyzing high-dimensional and high-collinear data [31]. It assumes that the target process or system is driven by a set of latent variables (LVs), which are extracted by searching for linear combinations of the input variables. The extreme learning machine (ELM) is a single hidden layer feedforward network with randomly generated input weights, while K-ELM is an extension of ELM that uses kernel functions instead of hidden layer feature mapping [32]; the ELM model has the advantages of rapid process speed: One reason is that the algorithm itself does not have backpropagation, and the other is that the hidden layer mapping is replaced with the kernel trip. Due to its fast computation speed and strong generalization ability, K-ELM is frequently used for both qualitative and quantitative analysis of spectral data [33,34].

To visualize the class separability of mineral samples, t-SNE was further adopted to project the high-dimensional data into a two-dimensional space. The common PCA model cannot effectively reflect the classification, while t-SNE is a nonlinear dimensionality reduction method that maintains the local data structure and presents the global distribution of samples [35]. We also used FS to select key variables in LIBS and RS data that contribute to the mineral identification. For each variable, FS maximizes the distance between elements of different categories and minimizes the distance between elements in the same category. In addition, the chemical elements related to these key variables were determined according to the NIST Atomic Spectra Database and the RRUFF Mineral Spectra Database [23].

The mineral samples were randomly divided into training and test samples in a ratio of 1:1 (25 spectra per sample stayed together). For each partition, we adjusted the parameters on the training set to obtain the optimized model, which was used to process the external test set. The whole process was repeated 50 times, and the average results of training and testing were used to evaluate the classification accuracy of PLS-DA and K-ELM. In the training set, the optimal parameters of PLS-DA and K-ELM were determined by 5-fold cross-validation. The optimal number of LVs in PLS-DA ranges from 1 to 10. In this work, the optimal number of LVs is 7. K-ELM adopts the radial basis function (RBF) kernel and selects the optimal kernel (C) and regularization parameter (γ) in the logarithmic scale range from 2^1^ to 2^20^. Data processing was carried out in MATLAB R2018b environment (The Mathworks Inc., Natick, MA, USA).

## 4. Conclusions

In this work, we used an integrated spectroscopic system and machine learning to identify mineral species. The integrated system combines LIBS and RS to enable the acquisition of chemical elements and molecular information of mineral samples. It obtained two different types of LIBS and RS spectra simultaneously, inheriting the high simplicity of spectra measurement. The spectral data were projected into low-dimensional space through t-SNE. Moreover, key spectral lines that contribute to mineral classification were identified by FS. The system was tested on six types of minerals. After pre-processing the obtained spectra, PLS-DA and K-ELM were used for classification.

The results show that PLS-DA and K-ELM combined with data fusion achieved the highest test accuracies of 97.7% and 98.4%, respectively, which demonstrates the feasibility of the integrated LIBS-RS system for mineral classification. Important spectral lines and their corresponding elements and bonds, such as lines of Al (I) (441.55 nm) and Ca-O (282 cm^−1^), indicate a clear separation of between-class intensities of mineral spectra. The experimental results demonstrate that the integrated LIBS-RS system combined with machine learning can be used as a fast and reliable analytical tool for geological exploration. We acknowledge the need to test our system on visually similar minerals from various localities with different impurity compositions and plan to address this in future research. This includes examining the impact of different impurities on mineral identification. Our future work will also focus on optimizing the experimental system and establishing a comprehensive database to further improve the efficiency of mineral identification.

## Figures and Tables

**Table 1:** Comparisons of PLS-DA on raw and pre-processed spectral data: classification accuracy.

	Data Sets	Raw Data	Pre-Processed Data
LIBS	Training	92.63% ± 3.78% ±3.78	94.10% ± 3.66% ±3.66
	Test	94.42% ± 4.70% ±4.70	95.61% ± 2.45% ±2.45
RS	Training	90.36% ± 4.39% ±4.39	93.34% ± 3.49% ±3.49
	Test	94.74% ± 5.47% ±5.47	95.61% ± 4.33% ±4.33

**Table 2:** The accuracy (%) and standard deviation (%) of PLS-DA and K-ELM on LIBS, Raman, and fusion data.

	Method	LIBS	Raman	Fusion
Training	PLS-DA	94.10 ±3.66	93.34 ± 3.49	96.12 ± 3.49
	K-ELM	97.12 ± 3.37	96.98 ± 3.19	97.67 ± 3.25
Test	PLS-DA	95.61 ± 2.45	95.61 ± 4.33	97.24 ± 1.44
	K-ELM	97.78 ± 1.53	97.73 ± 1.48	98.39 ± 1.27

## Data Availability

Data are contained within the article and supplementary materials.

## Supplementary Materials

The following supporting information can be downloaded at: https://www.mdpi.com/article/10.3390/molecules29143317/s1, Figure S1: The schematic diagram of the processing workflow of raw spectra; Table S1: Top 10 Significant Raman Peaks Data; Table S2: Top 10 Significant LIBS Peaks Data; Figure S2: The Fisher score of LIBS (a) and Raman (b) spectral data used for mineral classification; Figure S3: The intensity difference of key variables of LIBS (a) and RS (b) data; Table S3: The top 10 important variables of LIBS and RS data determined by Fisher score. Line (I) and (II) represents atomic emission and ion emission, respectively.
