# Supplementary material for: Fusion of Laser-Induced Breakdown Spectroscopy and Raman Spectroscopy for Mineral Identification Based on Machine Learning
Source: Molecules. 2024 Jul 14;29(14):3317. doi: 10.3390/molecules29143317 (PMC11279303; doi:10.3390/molecules29143317)
Supplement: Supplementary file 1 [file molecules-29-03317-s001.zip › molecules-3055820-supplementary.pdf]

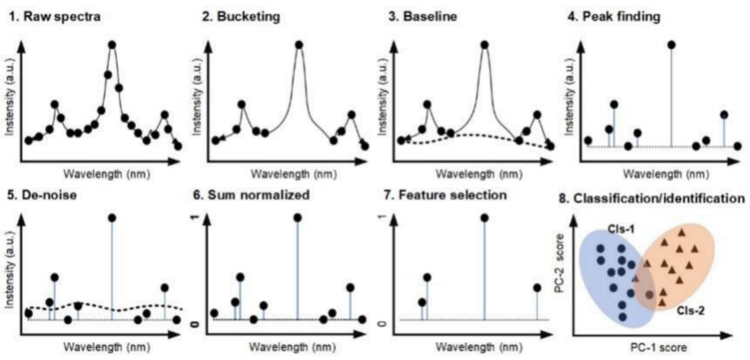

Fig. S1 The schematic diagram of the processing workflow of raw spectra.

Table S1. Top 10 Significant Raman Peaks Data.

	RS shift (cm^{-1})	Normalized Intensity(a.u.)		RS shift (cm^{-1})	Normalized Intensity(a.u.)		RS shift (cm^{-1})	Normalized Intensity(a.u.)
Cls-1	468	9000	Cls-2	278	5919	Cls-3	273	1644
	261	3000		466	3420		1420	1643
	402	3000		273	2401		716	1560
	132	2000		120	2090		278	1249
	278	1912		198	1876		1475	1108
	207	1500		289	1859		1294	1095
	716	1266		222	1695		170	700
	354	1000		414	1612		282	575
	282	902		282	455		141	566
	1030	330		1030	450		1030	239
	RS shift (cm^{-1})	Normalized Intensity(a.u.)		RS shift (cm^{-1})	Normalized Intensity(a.u.)		RS shift (cm^{-1})	Normalized Intensity(a.u.)
Cls-4	466	8828	Cls-5	278	5919	Cls-6	716	1700
	1303	8095		466	3420		273	1644
	203	2875		148	2918		278	1249
	117	2814		273	2401		148	918
	716	1450		127	1900		282	574
	352	932		289	1859		395	502
	266	876		716	1780		108	321
	1030	760		222	1695		1030	280
	395	712		282	455		124	263
	1164	510		1030	340		757	218

Table S2. Top 10 Significant LIBS Peaks Data.

	Wavelength (nm)	Normalized Intensity(a.u.)		Wavelength (nm)	Normalized Intensity(a.u.)		Wavelength (nm)	Normalized Intensity(a.u.)
Cls-1	249.07	2878	Cls-2	442.58	2649	Cls-3	403.3	7312
	254.96	693		403.3	2505		441.55	5702
	403.3	355		249.07	1786		249.07	5047
	442.58	315		441.55	1626		495.75	4767
	612.22	308		612.22	1463		254.96	4440
	441.55	282		254.96	1213		442.58	3216

	526.28	272		495.75	1050		489.18	2908
	495.75	260		489.18	790		526.28	782
	489.18	225		526.28	596		612.22	446
	458.14	216		458.14	570		458.14	333
	Wavelength (nm)	Normalized Intensity(a.u.)		Wavelength (nm)	Normalized Intensity(a.u.)		Wavelength (nm)	Normalized Intensity(a.u.)
Cls-4	442.58	2203	Cls-5	442.58	12960	Cls-6	442.58	16384
	612.22	1088		441.55	8174		441.55	11017
	249.07	1081		612.22	6981		612.22	8797
	403.3	1043		489.18	6776		489.18	8014
	441.55	1029		458.14	5584		458.14	6653
	254.96	692		526.28	2058		403.3	6373
	489.18	527		249.07	1612		526.28	2350
	458.14	447		403.3	862		249.07	1260
	526.28	430		254.96	650		254.96	1212
	495.75	379		495.75	319		495.75	814

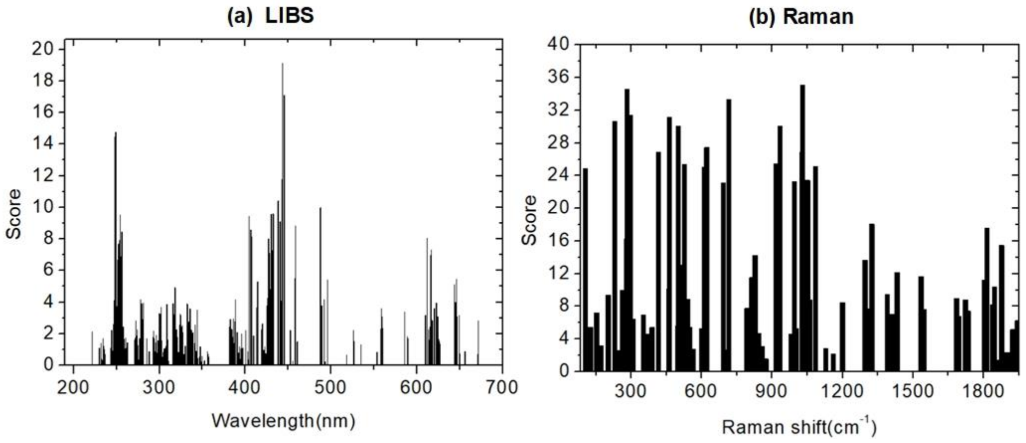

Fig. S2. The Fisher score of LIBS (a) and Raman (b) spectral data used for mineral classification.

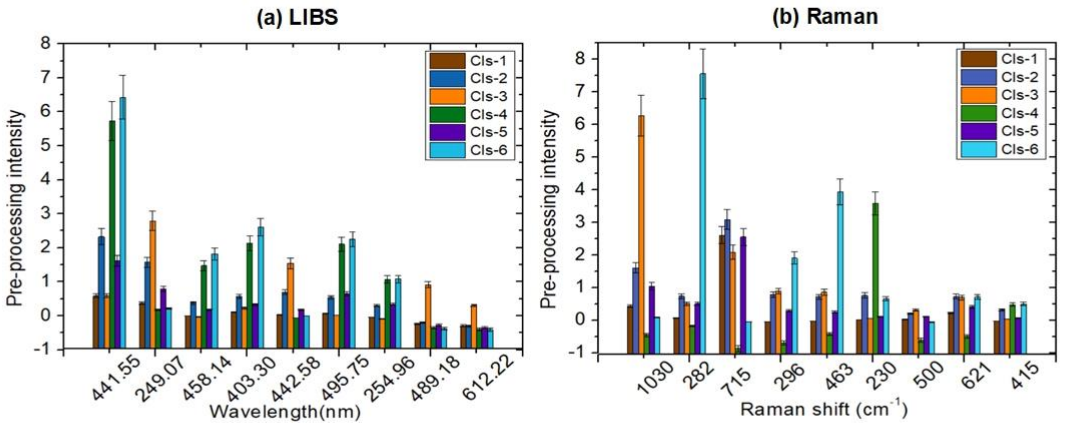

Fig. S3. The intensity difference of key variables of LIBS (a) and RS (b) data.

Table S3. The top 10 important variables of LIBS and RS data determined by Fisher score. Line (I) and (II) represents atomic

emission and ion emission, respectively.

**LIBS spectra**			**RS spectra**		
Element	Wavelength (nm)	Fisher score	bonds	RS shift (cm^{-1})	Fisher score
Al (I)	441.55	19.11	Si-O-Si	1030	35.05
Fe (I)	249.07	17.09	Ca-O	282	34.54
Ca (I)	458.14	14.73	CO_3^{2-}	715	33.36
Mn (I)	403.30	14.44	Fe-O	296	31.41
Ti (I)	442.58	13.86	Fe-O	463	31.13
Fe (I)	495.75	11.77	Fe-O	230	30.64
Fe (I)	254.96	10.54	Si-O	500	30.10
Ti (I)	489.18	10.40	SO_4^{2-}	621	27.45
Ca (I)	612.22	9.97	SO_4^{2-}	415	26.89
Fe (II)	526.28	9.59	Al-O-H	916	25.47
